# Dissociating object familiarity from linguistic properties in mirror word reading

**DOI:** 10.1186/1744-9081-3-43

**Published:** 2007-08-20

**Authors:** Alice M Proverbio, Friederike Wiedemann, Roberta Adorni, Valentina Rossi, Marzia Del Zotto, Alberto Zani

**Affiliations:** 1 Department of Psychology, University of Milano-Bicocca, Via dell'Innovazione 10, 20126 Milan, Italy; 2 Max Planck Institute for Brain Research, 60528 Frankfurt am Main, Germany; 3 Institute of Molecular Bioimaging and Physiology, CNR, 20090 Segrate, Milan, Italy

## Abstract

**Background:**

It is known that the orthographic properties of linguistic stimuli are processed within the left occipitotemporal cortex at about 150–200 ms. We recorded event-related potentials (ERPs) to words in standard or mirror orientation to investigate the role of visual word form in reading. Word inversion was performed to determine whether rotated words lose their linguistic properties.

**Methods:**

About 1300 Italian words and legal pseudo-words were presented to 18 right-handed Italian students engaged in a letter detection task. EEG was recorded from 128 scalp sites.

**Results:**

ERPs showed an early effect of word orientation at ~150 ms, with larger N1 amplitudes to rotated than to standard words. Low-resolution brain electromagnetic tomography (LORETA) revealed an increase in N1 to rotated words primarily in the right occipital lobe (BA 18), which may indicate an effect of stimulus familiarity. N1 was greater to target than to non-target letters at left lateral occipital sites, thus reflecting the first stage of orthographic processing. LORETA revealed a strong focus of activation for this effect in the left fusiform gyrus (BA 37), which is consistent with the so-called visual word form area (VWFA). Standard words (compared to pseudowords) elicited an enhancement of left occipito/temporal negativity at about 250–350 ms, followed by a larger anterior P3, a reduced frontal N400 and a huge late positivity. Lexical effects for rotated strings were delayed by about 100 ms at occipito/temporal sites, and were totally absent at later processing stages. This suggests the presence of implicit reading processes, which were pre-attentive and of perceptual nature for mirror strings.

**Conclusion:**

The contrast between inverted and standard words did not lead to the identification of a purely linguistic brain region. This finding suggests some caveats in the interpretation of the inversion effect in subtractive paradigms.

## Background

Neurofunctional studies have shown that the left midfusiform cortex (VWFA) responds with greater activation to linguistic than to non-linguistic stimuli and to real versus false fonts or non-letter strings [1-7]. For this reason, this area seems to play a crucial role in visual word form representation. ERP and MEG studies have identified a negative response peaking at about 150–200 ms (also named N170) as the electromagnetic manifestation of such activity [8-12].

We investigated the mechanism of orthographic analysis in silent reading by recording event-related potentials (ERPs) to words in standard or inverted orientation. The word rotation was performed to determine whether rotated items lose their linguistic properties. The inversion of an object's canonical orientation is especially used in subtractive neurometabolic paradigms to deprive familiar configurations (such as houses or faces) of their functional properties as visual entities, the overall luminance, colour and spatial frequency features being equal [13-15]. In this way, the presence of brain regions specifically responsive to familiar objects as unitary visual shapes, or belonging to distinct semantic categories (faces, hands, houses, cars, words), is investigated. A previous ERP study [16] compared inversion effects for three different stimulus categories (words, cars and faces). Stimulus inversion affected the latency of N170 for all kinds of visual objects resulting in a delayed response to inverted compared to standard orientations. The authors performed source modelling on the difference wave obtained by subtracting the waveforms to rotated stimuli from those to standard stimuli and found no difference in source localization as a function of stimulus category. The surface data were explained by two sources located in the left and right lateral occipital/fusiform gyrus areas. The amplitude of N170 was greater for words than cars in the left hemisphere, showing a typical pattern of orthographic sensitivity for the left lateral occipital areas. As for the inversion effects, N170 was not sensitive to word orientation at left sites, but greater to inverted than to standard words at right occipital areas. The authors did not specifically discuss this particular effect. To our knowledge, the Rossion et al. paper [16] is the only ERP study investigating the inversion effect with words rotated upside down; there has been no other ERP study of the effect of mirror words on reading.

On the other hand, a number of neuroimaging studies have explored the neural basis of mirror word reading to investigate visual skill learning or visual priming effects [17-19]. For example, Ryan and Schnyer [18] investigated the neural bases of format-specific priming in a mirror word-reading task using event-related fMRI and found that, while priming effects were greatest when the visual forms of primes and test words matched, mirror words were able to induce priming effects in standard word reading. This suggests the existence of format-invariant processes for visually presented words. The regions more sensitive to word orientation, and showing greater activity during reading of mirror than standard words, were confined primarily to the right hemisphere, namely the right superior temporal gyrus, anterior inferior frontal gyrus and middle frontal gyrus. These data agree with those of Poldrack and coworkers [19], comparing reading of mirror-reversed vs. normally-oriented text and showing a significant increase during mirror reading in the activation of the occipital cortex, right cuneus, and especially the right as opposed to left fusiform/lingual gyrus. Overall, this evidence, although compatible with the Rossion finding [16] of a larger inversion effect in the right lateral occipital area, is difficult to reconcile with the literature providing evidence of a strong inversion effect within the area devoted to the processing of a specific category (e.g. the face inversion effect is larger within the face fusiform area: see [20] for review).

The aims of our study were manifold. First of all, we aimed to investigate the timing and source localization of word processing and rotation effects by ERP analysis and LORETA modelling. In particular, it was not clear from previous literature whether word rotation affected the degree of activation of the left lateral occipital area (supposedly reflecting the underlying activity of the VWFA). Indeed, while it is widely agreed that left occipito/temporal N170 is affected by stimulus linguistic properties, being larger for letters than for non-orthographic symbols (e.g. [8,12,21-23]), not much is known about the effect of word inversion. For example, no inversion effect was observed by Rossion and coworkers [16] for N170 amplitude in the left lateral occipital areas, where the region of maximum surface activity during orthographic processing is usually located.

The second goal was to compare the effect of orthographic processing (observed by comparing ERPs to target and non-target letters) directly with that of string familiarity (obtained by comparing ERPs to standard and mirror words) in order to assess whether these effects overlapped somewhat or were independent at both the anatomical and functional levels. The assumption that the targetness effect (the difference between brain potentials or activity related to target minus those related to non-attended/searched letters) might be able to tell us something about orthographic processing comes from the shared assumption in Cognitive Neuroscience that the selective attention effect reflects the enhanced activity of visual areas generally devoted to the processing of a given stimulus feature example (e.g. the extrastriate area for spatial location, MT for motion, V4 for colour, parahippocampal area for places, VWFA for letter-strings). Likewise, the enhancement of neuronal activity during search or selection for specific letters (as unitary visual objects) should somewhat index the area devoted to orthographic processing. As a matter of fact, Flowers et al. [24] recently showed that attending to letters was associated with enhanced activity in a portion of the left extrastriate cortex, lateral to the visual word form area. Therefore, while some portions of the ventral extrastriate cortex are activated by attention to both alphabetic and non-alphabetic features, a letter-specific area was identified in Brodmann's Area 37.

Third, we aimed to investigate the extent to which the lexical properties of mirror words are eventually accessed and at which latency range; in other words, whether implicit reading effects (word/pseudoword ERP differences), commonly reported for standard words (e.g. [25,26]), are still observable with words in inverted orientations.

## Methods

Fifteen right-handed Italian students with right eye dominance (9 males and 9 females) participated in the present study. Their mean age was 22.2 (SD = 2.52). All had normal or corrected-to-normal vision and reported no history of neurological illness or drug abuse. Handedness was assessed by a laterality preference inventory [27] while eye dominance was determined by two independent practical tests. The data from two subjects were subsequently discarded before ERP averaging because of excessive eye movements. Experiments were conducted with the understanding and the written consent of each participant and in accordance with ethical standards (Helsinki, 1964). Subjects earned academic credits for their participation.

**Figure 1:**
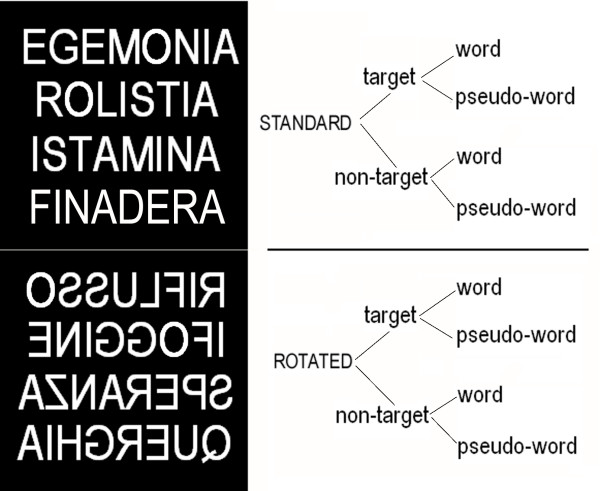
**Examplars of stimuli for each of the 8 categories**. In this example the target letter was O.

1280 linguistic strings (640 words and 640 legal pseudo-words) were randomly presented at the central visual field for 200 ms with an ISI varying between 1400 and 1600 ms. Stimuli were 45' in height, from 2° 15' to 4° in length, white on a black background in capital letters and Arial Narrow font. Half of them were presented in horizontally-inverted (i.e. mirror) orientation. Stimuli were balanced for length (5–9 letters), imageability, abstractness and frequency of use. Half the stimuli were targets, in that they contained a given target letter announced by the experimenter at the beginning of each run; the remainder were non-targets in that they did not include the target letter. Half of them appeared in standard orientation and the other half appeared horizontally-inverted (see Fig. 1). All stimuli were regularly pronounceable. The frequency distribution of consonants was the same in pseudo-words as in the real words. Target letters were selected that did not change their appearance in mirror orientation: they were A, H, I, M, O, T, U, V. Targets were also balanced for the position of the target letter within the string (beginning, middle or final part of string) and initial letter.

Participants sat comfortably in a darkened, acoustically and electrically shielded box in front of a computer screen located 114 cm from their eyes. They were instructed to fixate the centre of the screen and avoid any eye or body movements during the recording session.

The task consisted in responding to the target letter by pressing a button with the index finger of the left or right hand as accurately and rapidly as possible. The two hands were used alternately during the recording session, and the hand and sequence order were counterbalanced across subjects.

**Figure 2:**
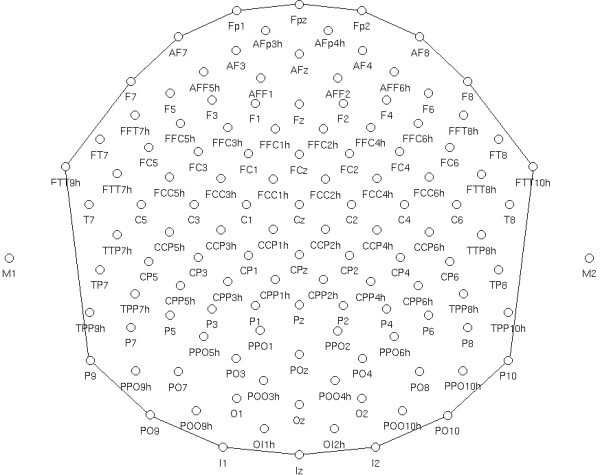
Schematic view of electrode montage (128 channels).

The EEG was continuously recorded from 128 scalp sites (see Fig. 2 for the complete electrode montage) at a sampling rate of 512 Hz. Horizontal and vertical eye movements were also recorded. Linked ears served as the reference lead. The EEG and electro-oculogram (EOG) were amplified with a half-amplitude band pass of 0.016–100 Hz. Electrode impedance was kept below 5 kΩ. EEG epochs were synchronized with the onset of stimuli presentation and analyzed by ANT-*EEProbe *software. Computerized artefact rejection was performed before averaging to discard epochs in which eye movements, blinks, excessive muscle potentials or amplifier blocking occurred. EEG epochs associated with an incorrect behavioural response were also excluded. The artefact rejection criterion was peak-to-peak amplitude exceeding 50 μV, and the rejection rate was ~5%. ERPs were averaged off-line from -200 ms before to 1000 ms after stimulus onset. The mean amplitudes of the N170 and N2 components of the ERPs were measured at the O1, O2, P7, P8, PPO9h and PPO10h electrode sites in the latency ranges 135–215 and 250–350 ms, respectively. Mean amplitude values were also measured at the same electrode sites in the time window 350–450 ms post-stimulus. The mean amplitude of N3/P3 was measured in the time window 470–570 ms at the left and right posterior temporal sites (P7, P8). The P300 area was measured at anterior sites (prefrontal: FP1, FP2; anterior frontal: AFF1, AFF2; and fronto/central: FC1, FC2) between 280 and 380 ms, while the anterior N400 area was measured at anterior sites (prefrontal: FP1, FP2; anterior frontal: AFF1, AFF2; and fronto/central: FC1, FC2) between 380 and 480 ms.

Response times exceeding mean ± 2 standard deviations were excluded. Behavioural and ERP data were subjected to multifactorial repeated-measures ANOVA. The factors were "orientation" (standard, rotated) and "response hand" (left, right) for RT data and additionally "letter targetness" (target, non-target), "electrode", (dependent on ERP component of interest) and "hemisphere" (left, right) for ERP data. Multiple comparisons of means were done by post-hoc Tukey tests.

Topographical voltage maps of ERPs were made by plotting colour-coded isopotentials obtained by interpolating voltage values between scalp electrodes at specific latencies. *Low Resolution Electromagnetic Tomography *(LORETA [28]) was performed on ERP difference waves at various time latencies using *ASA3 *and *ASA4 *software. LORETA, which is a discrete linear solution to the inverse EEG problem, corresponds to the 3D distribution of neuronal electric activity that has maximum similarity (i.e. maximum synchronization), in terms of orientation and strength, between neighbouring neuronal populations (represented by adjacent voxels). Source space properties were: grid spacing = 20 mm; Tikhonov regularization: estimated SNR = 3.

## Results

### Behavioural data

**Figure 3:**
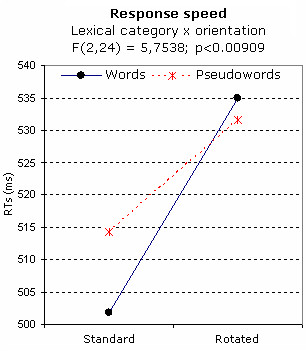
**Behavioural data**. Mean reaction times (N = 13) emitted in response to standard and rotated strings, as a function of their lexical category.

The analysis of response speed showed the significance of the orientation factor (F[1,12] = 89.48; p < 0.0001), with faster responses to stimuli in standard (506 ms) than in rotated (534 ms) orientation. The significant interaction of lexical category × orientation (F[2,24] = 5,754; p < 0.001) provided evidence of faster RTs in responding to words than to pseudo-words in standard orientation. The category factor was ineffective for rotated letter strings (see Fig. 3). Furthermore, responses were faster to standard than to rotated stimuli.

A significant interaction was found between orientation and response hand (F[1,12] = 13.91; p < 0.003), which showed faster RTs to standard stimuli with the right (500 ms) than the left (513 ms) hand. This difference was not observed for mirror stimuli (left = 534; right = 533 ms), as shown by post-hoc comparisons among means.

The ANOVA performed on the percentages of omitted responses (*arcsine *transformed) showed the significance of the orientation factor (F[1,12] = 172.17; p < 0.0001), revealing a greater percentage of omissions to rotated than to standard stimuli (standard = 6.52%; rotated = 15.25%). Considering that target letters (*per se*) did not change their appearance between the rotated and standard orientations, this effect reveals that words are treated as unitary visual shapes. Furthermore, the significance of lexical category (F[2,24] = 113.99; p < 0.0001) showed an overall larger number of omissions to pseudo-words than to words (words = 7.12%; pseudowords = 18.41%). The significant interaction between orientation and lexical category (F[2,24] = 4.90; p < 0.016) revealed, in both standard and rotated orientations, more omissions in pseudowords than words, especially when rotated. Indeed, the effect of orientation was more consistent for pseudo-words than for words.

Furthermore, for each lexical category, participants committed more omissions to rotated than to standard targets.

### Electrophysiological data

#### N170 component

**Figure 4:**
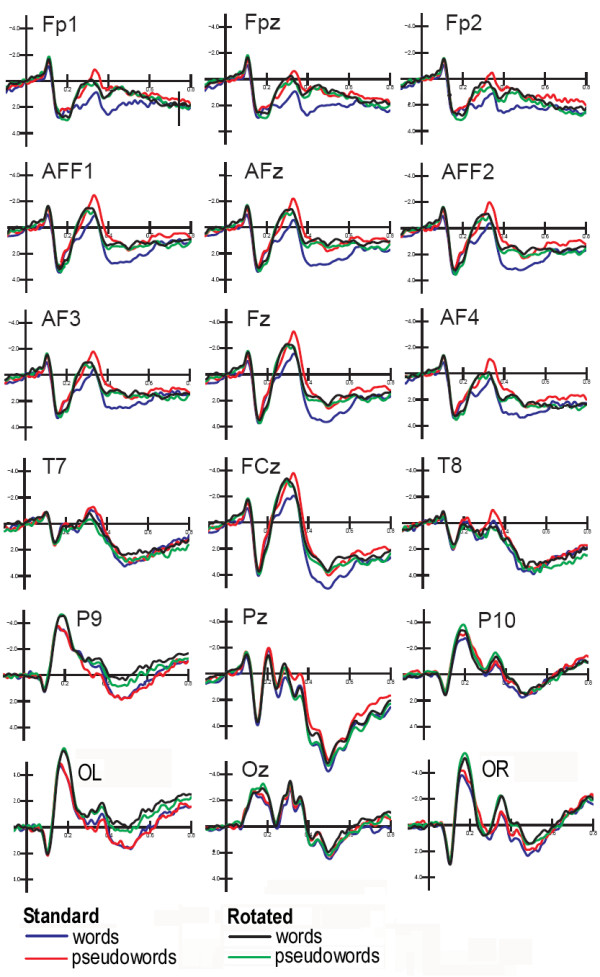
**Grand-average ERPs (N = 13) recorded in response to standard and rotated strings**. ERPs are shown as a function of stimulus lexical category but independently of target letter presence (targetness). Waveforms recorded from orbitofrontal, prefrontal (medial and lateral) frontal, central, anterior temporal, posterior temporal, lateral occipital and occipital electrode sites are displayed.

Grand-average ERPs recorded as a function of stimulus type are displayed in Fig. 4. An overview of the general pattern indicates that: (i) the P1 component was unaffected by lexical, orientation or target selection factors; (ii) stimulus orientation bilaterally affected the N1 component over the posterior-temporal/lateral occipital scalp sites independently of word meaning; (ii) lexical factors (word/pseudoword distinction) modulated left posterior brain activity as early as the N250 level, and anterior negativities and positive potentials were later and bilateral, thus revealing the presence of implicit reading processes mainly restricted to standard words in anterior brain areas.

**Figure 5:**
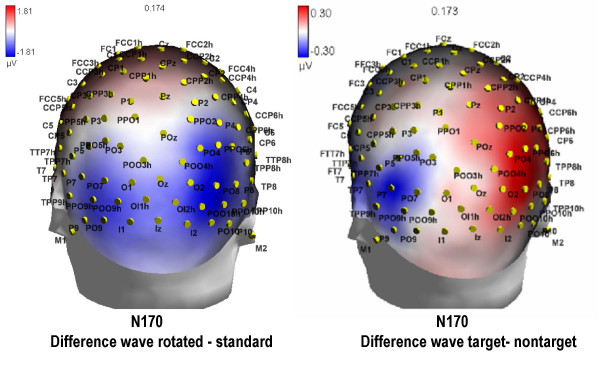
**Effect of orientation and letter selection at N1 level**. LEFT. Back view of topographical distribution of difference voltage (μV) obtained by subtracting grand-average ERPs to standard from ERPs to rotated words, at N1 peak. RIGHT. Topographical distribution of difference voltage obtained by subtracting grand-average ERPs to non-targets from ERPs to targets. A left-sided lateralization of the letter selection effect is evident, presumably linked to orthographic analysis.

The N1 component, measured at the mesial occipital (01/02, -6.49 μV), posterior temporal (P7/P8, -5.50 μV) and lateral occipital (PP09h/PP010h, -7.17 μV) sites, reached its maximum amplitude at about 170 ms at the lateral occipital sites as shown by the electrode factor (F[2,24] = 4.58, p < 0.021) and relative post-hoc comparisons. It was strongly sensitive to stimulus orientation (F[1,12] = 11.15; p < 0.006), with larger amplitudes to rotated (-6.67 μV) than to standard (-6.11 μV) stimuli as displayed in the topographic maps of Fig. 5 (Left).

LORETA [28] performed on the grand mean (N = 13) of the difference-wave (ERP responses to rotated minus standard words) in the N1 latency range (start 160, duration 20) showed a strong focus of activity in the right occipital lobe (BA 18) and a smaller focus in the left inferior occipital gyrus (BA19), as reported in Table 1. This effect might be due to a difference in stimulus familiarity for objects in a non-canonical orientation. Single source localization studies (LORETA) were performed for each individual to investigate inter-subject variability in the results obtained with the grand-mean waveforms.

**Table 1:** Tailarach coordinates corresponding to the intracranial generators explaining the difference voltages: Rotated minus standard between 160 and 180 ms (grand mean, N = 13), according to LORETA (ASA).

**Difference wave: Rotated-minus standard (160–180 ms)**				
**Power RMS 20.7 [μV]**				
**Magn.**	**T-x**	**T-y**	**T-z**	
	
**[nAm]**	**[mm]**	**[mm]**	**[mm]**	**Hemisphere, Lobe, BA**

0.393	18.6	-5.4	-12.4	Right Cerebrum, Limbic Lobe, Parahippocampal G.,
0.086	-38	30	43	Left Cerebrum, Frontal Lobe, Middle Frontal G., BA 8
0.072	0	56	5	Left Cerebrum, Frontal Lobe, Medial Frontal G., BA 10
0.101	0	52	44	Right Cerebrum, Frontal Lobe, Medial Frontal G., BA 8
0.078	19	-5	-12	Right Cerebrum, Frontal Lobe, Middle Frontal G., BA 8
**0.524**	-38.9	-68.1	-3.8	Left Cerebrum, Occipital Lobe, Inferior Occipital G., BA 19
**0.719**	37.7	-68.1	-4.6	Right Cerebrum, Occipital Lobe, Lingual G., BA18

In the anterior brain areas, all subjects (N = 13) showed activation of the left middle frontal gyrus (BA9/10), whereas 9 out of 13 also exhibited activation of the right middle or superior frontal gyrus (BA 9/10). In the posterior brain regions, 6 subjects showed activation of the left middle temporal gyrus (BA37/21), 9 of the left occipital lobe (BA 18/19), 9 of the right occipital lobe (fusiform gyrus, BA18), and 6 of the right inferior temporal lobe/middle temporal lobe (BA20/21). Table 2 summarizes the relative inter-individual variability in the localization of the effect, showing overall a bilateral activation of the occipito/temporal regions, which was strongly right-lateralized in 7 (out of 13) individuals. By considering only the common activations in the right hemisphere for the rotated minus standard difference voltage between 160 and 180 ms, and computing the mean T-x, T-y, T-z coordinates across 9 subjects, a mean source in the right occipital lobe (fusiform gyrus, BA18) was obtained with a magnitude of 1.283 nAm. Using the same procedure, a mean source in the occipital lobe (fusiform gyrus, BA19) with a magnitude of 0.966 nAm was obtained by observing the regions of common activation in the left hemisphere across 9 subjects. The individual data are strongly compatible with the results obtained from the grand-mean difference waves.

**Table 2:** Areas of common activation (N = 9) for the difference voltage: Rotated minus standard between 160 and 180 ms are listed along with their anatomical localizations, according to LORETA (ASA)

**Right Hemisphere**										
**Ss.**	**Magn [nAm]**	**Power RMS**	**T-x [mm]**	**T-y [mm]**	**T-z [mm]**	**Hem.**	**Lobe**	**Area**	**BA**	**[mm]**

AB	1.582	47.1	38	-65	-9	RH	Occipital	Fusiform G.	BA 19	Range = 6
AP	1.691	42.1	38	-65	-9	RH	Occipital	Fusiform G.	BA 19	Range = 6
DT	0.347	16.3	38	-65	-9	RH	Occipital	Fusiform G.	BA 19	Range = 6
DV	1.079	31.1	38	-65	-9	RH	Occipital	Fusiform G.	BA 19	Range = 6
ES	1.183	28.8	38	-65	-9	RH	Occipital	Fusiform G.	BA 19	Range = 6
GU	1.753	40.8	19	-87	-10	RH	Occipital	Fusiform G.	BA 18	Range = 7
MDB	0.946	45.6	19	-87	-10	RH	Occipital	Fusiform G.	BA 18	Range = 7
SM	0.289	17.9	19	-89	3	RH	Occipital	Lingual G.	BA 17	Range = 9
ST	2.676	59.1	38	-65	-9	RH	Occipital	Fusiform G.	BA 19	Range = 6
Mean	**1.283**		31.7	-72.5	-7.89	**RH**	**Occipital**	**Fusiform G.**	**BA 18**	3 mm
SD	0.703		13	26	10					

**Left hemisphere**										

**Ss.**	**Magn [nAm]**	**Power RMS**	**T-x [mm]**	**T-y [mm]**	**T-z [mm]**	**Hem.**	**Lobe**	**Area**	**BA**	**[mm]**

DT	0.386	16.3	-38	-65	-9	LH	Occipital	Fusiform G.	BA 19	Range = 7
DV	0.743	31.1	-38	-65	-9	LH	Occipital	Fusiform G.	BA 19	Range = 7
EB	1.618	39.5	-38	-65	-9	LH	Occipital	Fusiform G.	BA 19	Range = 7
ES	0.395	28.8	-31	-43	-8	LH	Temporal	Fusiform G.	BA 37	Range = 9
GPB	0.414	21.2	-19	-87	-10	LH	Occipital	Lingual G.	BA 18	Range = 1
LDB	1.212	38.2	-19	-87	-10	LH	Occipital	Lingual G.	BA 18	Range = 1
MC	0.352	11.6	-38	-65	-9	LH	Occipital	Fusiform G.	BA 19	Range = 7
MDB	1.926	45.6	-38	-65	-9	LH	Occipital	Fusiform G.	BA 19	Range = 7
SM	0.662	17.9	-38	-65	-9	LH	Occipital	Fusiform G.	BA 19	Range = 7
ST	1.956	59.1	-38	-65	-9	LH	Occipital	Fusiform G.	BA 19	Range = 7
Mean	**0.9664**	33.8	-33.5	-67.2	-9	**LH**	**Occipital**	**Fusiform G.**	**BA 19**	3 mm
SD	0.62238		7.54	11.8	0.56					

**Figure 6:**
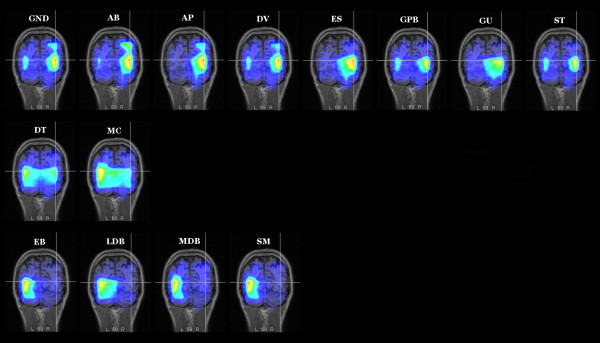
**LORETA: Source localization for the word rotation effect**. For each individual, LORETAs were performed on the ERP difference-wave of rotated minus standard words in the N1 latency range (start 160, duration 20). Both grand mean LORETA (label = GND) and individual solutions show a strong focus of activity (in nAm) in the right occipital gyrus (BA 18, lingual/fusiform gyrus).

Fig. 6 shows an overview of the results obtained for the rotated-standard comparisons, along with the grand mean LORETA. The cross lines indicate the exact coordinates of maximum strength of the right focus for the grand-mean LORETA, and are perfectly compatible with the focus exhibited by 7 individuals (strongly lateralized), a further 2 subjects at bilateral sites, and lastly 4 subjects in the homologous left location.

**Figure 7:**
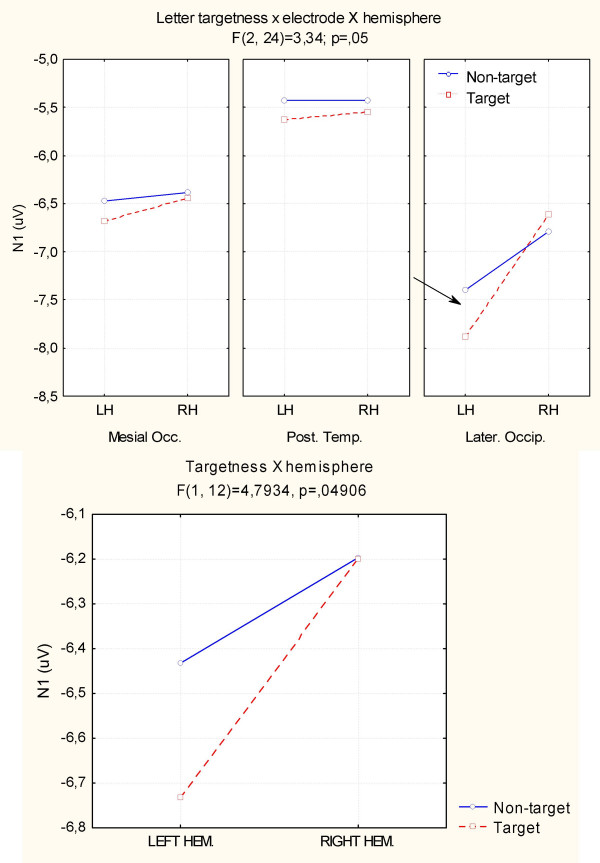
**Targetness × hemisphere interaction for N1 component**. ANOVA results and post-hoc comparisons showed a strong left hemispheric lateralization for the letter selection effect.

ANOVA also revealed an interaction between letter targetness and hemisphere (F[1,12] = 4.80; p < 0.049), showing a significant target/non-target difference in the response recorded over the left but not the right hemisphere. Also significant was the interaction of target × electrode × hemisphere (F[2,24] = 3.33; p = 0.05). Post-hoc comparisons indicated anatomical specificity for the effect of orthographic selection (see Fig. 7), with larger N1 responses to target at left lateral-occipital sites. This effect is clearly visible in Fig. 5 (Right), which shows the left-sided topographical distribution of the negativity elicited by target letters obtained by subtracting grand-average ERPs to non-targets from ERPs to targets between 160 and 180 ms, the time window corresponding to the N1 peak.

**Figure 8:**
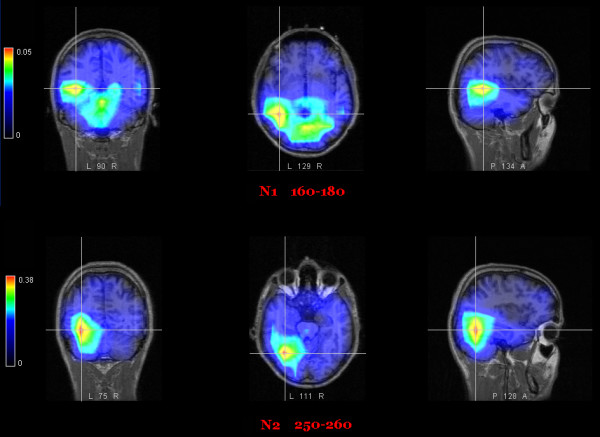
**LORETA: Source localization for the letter recognition effect (orthographic analysis)**. **Top**: LORETA performed on the grand-mean target/non-target difference-wave between 160 and 180 ms (grid spacing = 20, ratio = 3) showing a strong focus of activation (in nAm) in the left fusiform gyrus (BA 37; x = -43, y = -57, z = 3). Bottom: same thing for N2 component. LORETA performed on the target/non-target difference-wave between 250 and 260 (grid spacing = 20, ratio = 3) showing a much stronger focus of activation in the left fusiform gyrus of the temporal lobe (BA 37, x = -29, y = -43, z = -14).

A LORETA performed on the difference-wave obtained by subtracting ERPs to non-targets from ERPs to targets in the time window corresponding to the N1 peak (160–180) showed a strong focus of activation for this effect in the left fusiform gyrus (BA 37; x = -43, y = -57, z = 3) as listed in Table 3. The grand average LORETA solution is shown in Fig. 8 (upper row). In addition, single source localization studies (LORETA) were performed for each individual in the same latency range to investigate inter-subject variability in the results obtained from the grand-mean difference wave.

**Table 3:** Tailarach coordinates corresponding to the intracranial generators explaining the difference voltage: Target minus non-target between 160 and 180 ms (grand mean, N = 13), according to LORETA (ASA).

**Difference wave: Target – non target**				
**Power RMS 4.5 [μV]**				
**Magn.**	**T-x**	**T-y**	**T-z**	
	
**[nAm]**	**[mm]**	**[mm]**	**[mm]**	**Hemisphere, Lobe, BA**

0.039	-39.4	38.3	41.7	Left Frontal Lobe, Middle Frontal G., BA 8
0.059	33.4	57.5	14.3	Right Frontal Lobe, Superior Frontal G., BA 10
0.044	49.9	0	-30.8	Right Temporal Lobe, Middle Temporal G., BA 21
**0.086**	-43.7	-57.5	3	Left Temporal lobe, Middle Temporal G., BA 37 (FG)
**0.079**	31.5	-57.5	-3.5	Right Cerebrum, Occipital Lobe, Lingual G., BA 19

In the anterior brain regions, 11 out of 13 subjects exhibited activation of the left middle frontal gyrus (x = -38, SD = 0; y = 28, SD = 0; z = 32, SD = 0; BA 9), one showed activation of the left superior frontal gyrus (x = -38; y = 52; z = 15; BA10) and seven showed activation of the medial frontal gyrus (x = 0, SD = 0; y = 50, SD = 0; z = 28, SD = 12; BA9). In the right frontal region, 9 out of 13 subjects exhibited activation of the right middle frontal gyrus (x = 38, SD = 0; y = 28, SD = 0; z = 32, SD = 0; BA 9), one of the right superior frontal gyrus (x = 38; y = 52; z = 15; BA10), and two of the right precentral gyrus (x = 57, SD = 0; y = 8, SD = 0; z = 11, SD = 0; BA 44).

Posteriorly, 8 out of 13 subjects showed activation of the left middle temporal gyrus (BA37/21, magnitude 0.300), 8 of the left superior temporal gyrus (BA 38), and 8 of the left lingual/fusiform gyrus of the occipital lobe (BA18/19). As for the right posterior brain regions, all subjects (N = 13) exhibited activation of the right middle temporal gyrus (BA 21, x = 57, SD = 0; y = -6, SD = 0; z = -17, SD = 0). Three subjects showed further activation of the right middle temporal gyrus/superior occipital gyrus BA39 (x = 38, SD = 0; y = -69, SD = 0; z = 23, SD = 0), another 5 of the right middle temporal gyrus BA 20 (x = 57, SD = 0; y = -43, SD = 0; z = -8, SD = 0), and the remaining 5 of the right lingual gyrus of the occipital lobe (BA18/19) (x = 31, SD = 9; y = -73, SD = 11; z = -9, SD = 0.5). Table 4 shows the relative inter-individual variability in the localization of the effect, revealing overall an area of common activation for 10 subjects localized in the right middle temporal gyrus (BA21, magnitude = 0.245 nAm) and over the left fusiform gyrus of the temporal lobe (BA37, magnitude = 0.271 nAm).

**Table 4:** Areas of common activation (N = 10) over the occipito/temporal area for the difference voltage: Target-non-target between 160 and 180 ms are listed along with their anatomical localizations, according to LORETA (ASA)

**Right Hemisphere**										
**Ss.**	**Magn. [nAm]**	**Power RMS**	**T-x [mm]**	**T-y [mm]**	**T-z [mm]**	**Hem.**	**Lobe**	**Area**	**BA**	**[mm]**

AB	0.265	13.8	57.5	-6.5	-16.8	RH	Temporal	Middle Temp. G.	BA21	Range = 1
AP	0.19	12.5	57.5	-6.5	-16.8	RH	Temporal	Middle Temp. G.	BA21	Range = 1
DT	0.131	11.1	57.5	-6.5	-16.8	RH	Temporal	Middle Temp. G.	BA21	Range = 1
DV	0.28	16.6	57.5	-6.5	-16.8	RH	Temporal	Middle Temp. G.	BA21	Range = 1
EB	0.144	11.8	57.5	-6.5	-16.8	RH	Temporal	Middle Temp. G.	BA21	Range = 1
ES	0.562	22.9	57.5	-6.5	-16.8	RH	Temporal	Middle Temp. G.	BA21	Range = 1
GBP	0.261	9.8	57.5	-6.5	-16.8	RH	Temporal	Middle Temp. G.	BA21	Range = 1
LDB	0.141	10.2	57.5	-6.5	-16.8	RH	Temporal	Middle Temp. G.	BA21	Range = 1
SM	0.332	9.9	57.5	-6.5	-16.8	RH	Temporal	Middle Temp. G.	BA21	Range = 1
ST	0.172	13.3	57.5	-6.5	-16.8	RH	Temporal	Middle Temp. G.	BA21	Range = 1
Mean	**0.245**	13.2	57.5	-6.5	-16.8	**RH**	**Temporal**	**Middle Temp. G.**	**BA21**	1 mm
SD			0	0	0					

**Left hemisphere**										

**Ss.**	**Magn. [nAm]**	**Power RMS**	**T-x [mm]**	**T-y [mm]**	**T-z [mm]**	**Hem.**	**Lobe**	**Area**	**BA**	**[mm]**

AB	0.366	13.8	-19.2	-86.5	-9.7	LH	Occipital	Lingual G.	BA18	Range = 1
AP	0.258	12.5	-57.5	-42.6	-7.7	LH	Occipital	Fusiform G.	BA19	Range = 4
DT	0.245	11.1	-38.3	-64.5	-8.7	LH	Occipital	Fusiform G.	BA19	Range = 7
DV	0.193	16.6	-57.5	-42.6	-7.7	LH	Temporal	Middle Temp. G.	BA37	Range = 4
EB	0.22	11.8	-38.3	-64.5	-8.7	LH	Occipital	Fusiform G.	BA19	Range = 7
ES	0.375	22.9	-57.5	-42.6	-7.7	LH	Temporal	Middle Temp. G.	BA37	Range = 4
GPB	0.173	9.8	-19.2	-86.5	-9.7	LH	Occipital	Lingual G.	BA18	Range = 1
LDB	0.212	10.2	-38.3	-64.5	-8.7	LH	Occipital	Fusiform G.	BA19	Range = 7
SM	0.214	9.9	-57.5	-42.6	-7.7	LH	Temporal	Middle Temp. G.	BA37	Range = 4
ST	0.463	13.3	-38.3	-64.5	-8.7	LH	Occipital	Fusiform G.	BA19	Range = 7
Mean	**0.271**	13.2	-42	-60	-8	**LH**	**Temporal**	**Fusiform G.**	**BA37**	7 mm
SD			14	16	0.7					

#### Posterior N2 area (250–350)

**Figure 9:**
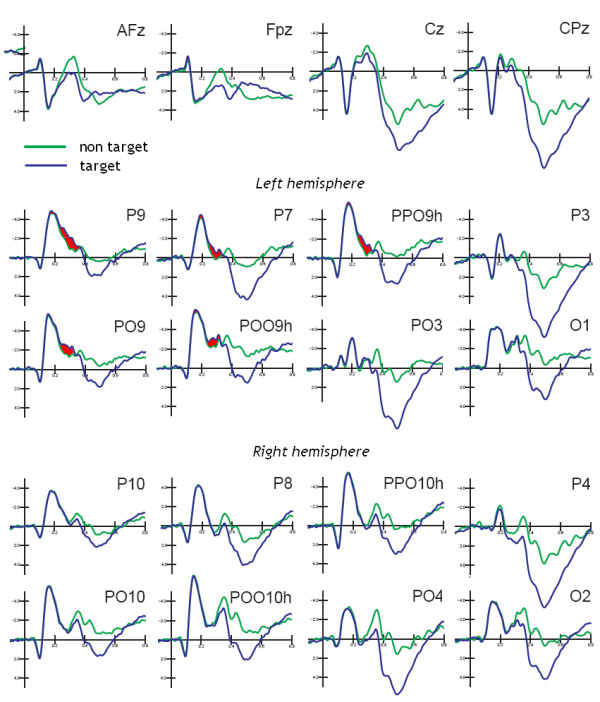
**Grand-average ERPs (N = 13) recorded in response to target and non-target stimuli**. ERPs are shown as a function of stimulus targetness, but independently of lexical or orientation factors. Waveforms displayed were recorded from midline anterior frontal, prefrontal, central, centro-parietal, and separately for left (Top) and Right (Bottom) hemisphere of recording, from posterior temporal, lateral occipital, ventral lateral occipital, occipito/parietal, mesial occipital electrode sites.

N2 deflection reached its maximum amplitude at the mesial occipital sites, as confirmed by the statistical significance of the electrode factor (F[2,24] = 8.31; p < 0.001) and relative post-hoc comparisons. It was also very sensitive to string orientation as indicated by the significance of the orientation factor (F[1,12] = 6.52; p < 0.02). As for N1, N2 was much greater to rotated (-1.05 μV) than to standard (-0.54 μV) stimuli. Again, N2 was affected by letter targetness but also by interaction with hemisphere (F[1,12] = 6.22; p < 0.02), being much larger to targets than to non-targets in the left hemisphere (target = -1.421; non-target = -0.895 μV), as clearly visible in Fig. 9 and shown by post-hoc comparisons.

The significant interaction of targetness × electrode × hemisphere (F[2,24] = 4.57, p < 0.02), and relative post-hoc comparisons among means, provided evidence of a greater effect of targetness at the left lateral occipital (PP09h, target = -1.16, non-target = -0.43 μV) and posterior temporal (P7, target = -0.87, non-target = -0.28 μV) sites compared to all other electrode locations.

Grand-average ERPs recorded as a function of stimulus targetness and independently of stimulus orientation or lexical factors are shown in Fig. 9. The shaded red area identifies the regions corresponding to selection negativity related to target selection, which was strongly focused over the left occipito/temporal area at both the N1 and N2 levels.

A LORETA performed on the difference-wave obtained by subtracting ERPs to non-targets from ERPs to targets in the time window corresponding to the N2 peak (250–260 ms) showed a strong focus of activation for this effect in the left fusiform gyrus of the temporal lobe (BA 37; x = -29, y = -43, z = -14), which is fully compatible with the localization of the VWFA (see Fig. 8, lower row).

**Figure 10:**
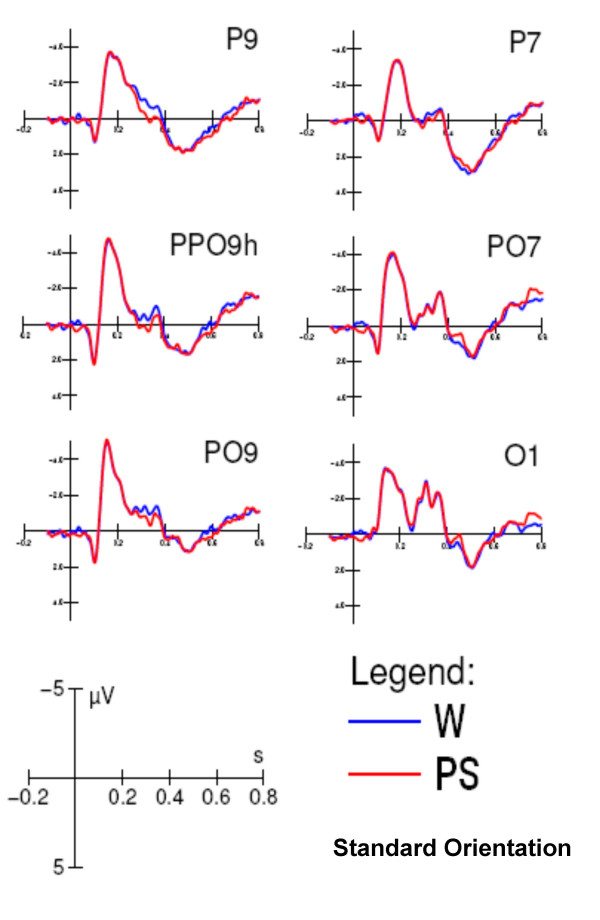
**The effect of lexical category of standard words at left posterior sites**. Grand-average ERPs (N = 13) recorded in response to words and pseudowords. The lexical effect was observable as a larger N2 response (250–350) in response to real words

The interaction of lexical category × orientation × electrode (F[2,24] = 5,1096; p < 0.0142), showed the presence of a larger N2 in response to words than to pseudo-words in standard (but not rotated) orientation at the lateral occipital sites (see Fig. 10). Indeed, words were not discriminated from pseudo-words in mirror orientation at this latency stage.

The further interaction of letter targetness × electrode × hemisphere (F[2,24] = 4.57; p < 0.02) revealed a strong hemispheric asymmetry for target-related negativity at each electrode site, but especially the lateral occipital ones, as confirmed by post-hoc comparisons. At this latency range we observed a strong tendency toward significance (F[2,24] = 3.23; p < 0.057 μV) for the orientation × electrode factor. Indeed, post-hoc comparisons showed the largest difference in N2 amplitude as a function of stimulus orientation at the mesial occipital sites.

#### Posterior N3/P3 area (350–450 ms)

In the next time window, ANOVA showed the significance of orientation (F[1,12] = 5.6; p < 0.04), with a greater N3 to rotated than to standard stimuli (standard = -0.303; rotated = -0.400 μV). Also significant was the interaction of letter targetness × lexical category (F[1,12] = 9.30; p < 0.02). Post-hoc comparisons showed larger positivities (ascending phase of P3) to targets than to non-targets for both words (non-target = -0.70; target = 0.63 μV) and pseudo-words (non-target = -0.5; target = 0.36 μV).

Again, targetness interacted with stimulus orientation (F[1,12] = 8.85; p < 0.01). Post-hoc comparisons showed greater positivities to targets than to non-targets, greater increases in positivities to targets in standard (1.04 μV) than rotated (-0.04 μV) orientation, and a lack of orientation effect for non-target stimuli (target = -0.43; non-target = -0.76 μV).

#### Posterior-temporal N3/P3 area (470–570 ms)

**Figure 11:**
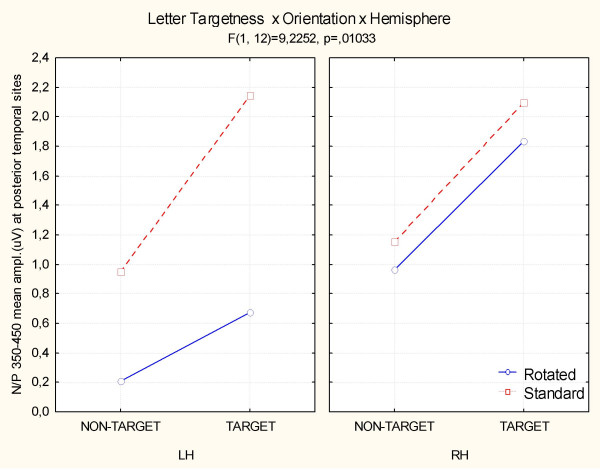
**N/P 350–450 Letter targetness × Orientation × Hemisphere**. Interaction between letter selection and word orientation between 350 and 450 ms at posterior/temporal sites. At this stage the right hemisphere was insensitive to word orientation during letter selection.

**Figure 12:**
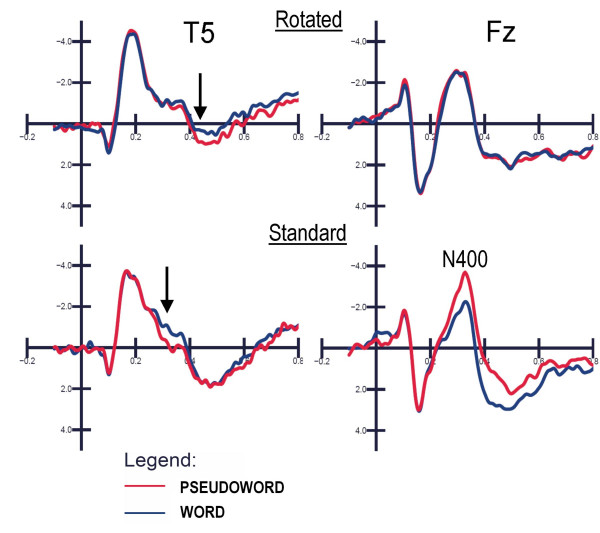
**Lexical access for rotated words is delayed by 100 ms but not disrupted**. Grand-average ERPs (N = 13) recorded in response to rotated and standard words as a function of their lexical category. Waveforms were recorded from the left posterior temporal and midline frontal sites. Later N4 and P3 lexical effects were not observable at anterior sites for mirror words. This suggests that implicit (pre-attentive) reading was of purely perceptual nature for mirror words.

ANOVA performed on the mean voltage recorded at the left and right posterior temporal sites revealed that the following were significant: letter targetness (F[1,12] = 11; p < 0.007), with greater positivity to targets (1.7 μV) than to non-targets (0.8 μV); lexical category (F[1,12] = 5.3; p < 0.05), with a greater positivity to pseudowords (1.4 μV) than to words (1.1 μV); orientation (F[1,12] = 12.1; p < 0.005), with a much greater positivity to standard (1.6 μV) than to rotated (0.9 μV) strings; and orientation × hemisphere (F[1,12] = 12.1; p < 0.005), with a strong left hemispheric lateralization for the orientation effect. The interaction of letter targetness orientation × hemisphere F[1,12] = 9.2; p < 0.01) and relative post-hoc comparisons revealed a much larger orientation effect for targets over the left hemispheric sites (p < 0.01), as shown in Fig. 11. Fig. 12 displays ERP waveforms recorded at the left posterior temporal and medial frontal sites as a function of stimulus orientation and lexical category. The arrow indicates the emergence of the lexical effect for rotated words, in the form of a larger negativity to words than to pseudowords at the left posterior temporal sites in the N3 latency range, which corresponds to a delay of about 100 ms compared to standard words.

#### Anterior P3 area (280–380 ms)

The P300 area measured at anterior sites (prefrontal: FP1, FP2; anterior frontal: AFF1, AFF2; and fronto/central: FC1, FC2) revealed significant effects of electrode (F[2,24] = 16.35, p < 0.0001), hemisphere (F[1,12] = 15.19, p < 0.002), and electrode × hemisphere (F[2,24] = 5.19, p < 0.01), with maximum amplitude bilaterally at prefrontal sites and a smaller but asymmetrically distributed positivity at the fronto/central sites (greater over the right hemisphere).

The significance of targetness (F[1,12] = 31.35, p = 0.0001) indicated a greater P300 response to targets (-0.3 μV) than to non-targets (1.54 μV), while the lexical category factor (F[1, 12] = 18.09, p < 0.001) indicated greater responses to words (-0.65 μV) than to pseudowords (-0.22 μV). The interaction of stimulus targetness with orientation (F[1, 12] = 8.516, p < 0.013) indicated no effect of orientation (difference between standard and rotated) for items including target letters, and a much greater target/non target difference (Δ) for standard (Δ = 1.61 μV, p < 0.0002) than rotated (Δ = 0.81, p < 0.01) words.

**Figure 13:**
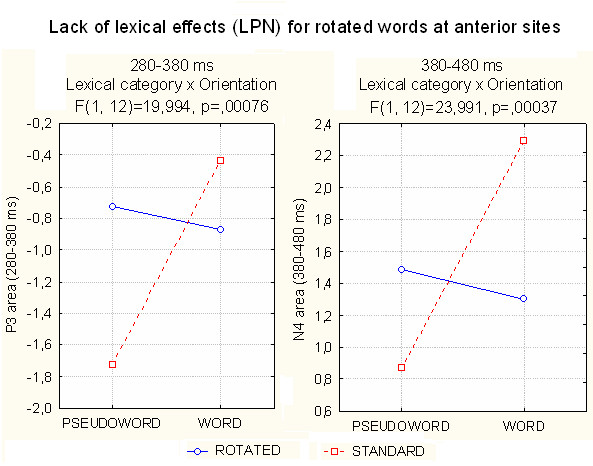
**Lexical category × orientation at frontal sites**. Both analyses of P3 (left) and N4 area (right) showed a lack of lexical effect (i.e. increased lexical processing negativity to pseudowords compared to words) for mirror words.

At this latency range, the significant interaction of lexical category × orientation (F[1,12] = 20, p < 0.0008) and relative post-hoc comparisons indicated no lexical effects for rotated words (see waveforms of Fig. 11) and greater P3 to words than pseudowords in standard orientation (p < 0.0007), as shown in the left part of Fig. 13.

#### Anterior N4 area (380–480)

The N400 area measured at anterior sites (prefrontal: FP1, FP2; anterior frontal: AFF1, AFF2; and fronto/central: FC1, FC2) revealed the significance of the lexical factor (F[1,12] = 20.47, p < 0.0001), indicating a much larger negativity to pseudowords (1.17 μV) than to words (1.8 μV). For this reason, this response might be assimilated into a lexical processing negativity, a sort of anterior N400 very sensitive to word familiarity and frequency. The interaction of Lexical category × Orientation (F[1,12] = 24, p < 0.0004) provided evidence of a null lexical effect (word/pseudoword difference) for rotated words at anterior sites, quite similar to what was found in the previous temporal window (see Fig. 13, right).

Lastly, the interaction of Letter targetness × Orientation (F[1,12] = 5, p < 0.04) and relative post-hoc comparisons evidenced a significant target effect for standard (target = 1.84; non-target = 1.3 μV) but not rotated (target = 1.3; non-target = 1.46 μV) strings.

## Discussion

The present findings have shown that letter selection (presumably linked to orthographic processing) specifically activates the left occipital/temporal area (BA37) as early as 170 ms post-stimulus, an area that is anatomically compatible with the VWFA, i.e. the region described in the literature as being devoted to letter and word processing [1-7]. This effect is fully consistent with the findings of Flowers et al. [24], who showed that attending to alphabetic (vs. non-alphabetic) characters resulted in enhanced activity of the left extrastriate area (BA37). These data suggest that attending to and searching for a target letter strongly activates neural populations normally devoted to letter reading and recognition.

On the other hand, the effect of word rotation, obtained by subtracting brain activation to standard from that to inverted words (as in subtractive neuroimaging paradigms), did not lead to the identification of a purely linguistic brain region. Instead, a visual area possibly sensitive to object familiarity, namely the right middle occipital gyrus (BA 18/19), showed the strongest sensitivity to words in a non-canonical orientation in the grand mean LORETA, and showed the strongest activation in the majority of subjects.

N170 was larger in amplitude to rotated (unfamiliar) than to standard words. Other neuroimaging studies have provided evidence of a reduced activation of object responsive areas in the ventral occipito-temporal cortex for more familiar than for unfamiliar objects [29], faces [30], words [31] and ideograms [32].

As for the greater activation of the right occipital lobe in response to rotated words, these findings are consistent with recent fMRI data showing increased activity of right hemispheric regions such as the right ventral-temporal areas [17], right-superior temporal gyrus [18], right occipital cortex and right fusiform/lingual gyrus [19] during reading of mirror vs. standard words.

Importantly, our results suggest some caveats about the use of inversion paradigms (with houses, faces or words) in the study of category-specific functional specialization of brain areas. Indeed, in the present experiment, while letter selection was strongly lateralized (obviously because of involved regions devoted to orthographic processing, such as the VWFA), the effect of word orientation was not. However, the possibility that this discrepancy only applies to linguistic objects cannot be excluded, therefore further investigation is needed before reaching a definitive conclusion.

In this regard, it may be of some interest to compare the effects related to word inversion with the large and complex literature on face structure processing making use of the face inversion paradigm. The face-inversion effect (i.e. the observation that faces are surprisingly more difficult to recognize when turned upside down than right side up) has been widely studied since its discovery by Yin [33]. The dominant explanation of this effect is that the human visual system's strategy for facial representation is primarily 'configural', i.e. it involves encoding the second-order spatial relationships between face parts such as eyes, nose and mouth [34,35]. Configural analysis is believed to be compromised in vertically inverted faces. While there is no doubt that this effect exists, and is also easily observable during infancy [36], there is no proof that upright and inverted faces are not processed by the same populations of neurons within the occipito/temporal cortex. Indeed, a specific brain area has been described (named the face fusiform area, FFA) that seems to respond more strongly to faces than to non-face objects (e.g. [37]).

The literature is not completely consistent about the significance of the face inversion effect with regard to FFA activity. Indeed, some fMRI studies showed either no [38,39] or a weak [40] face inversion effect in the FFA. This means the FFA may respond with the same amplitude to upright and inverted faces, so the difference between the two conditions would never lead (in this case) to the identification of FFA as the area responsible for face structure analysis.

Again, both Aguirre et al. [38] and Epstein et al. [41] found that inversion of face stimuli had no effect on the magnitude of responses in the fusiform face area, while inverted faces evoked greater neural responses than upright faces within object regions. Therefore, it might not be always true that in the object/face literature the strongest inversion effects are typically found at the sites in which category differences occur.

Some congenital prosopagnosia studies have shown a lack of face inversion effects in such patients [42], suggesting a close relationship between the presence of an inversion effect and the integrity of visual areas devoted to face processing. However, other prosopagnosic patients failed to show such a pattern. For example, Riesenhuber et al. [43] found no difference between the inversion effects of 'featural' (i.e. some detail such as the eye or the mouth) and 'configural' (i.e. the total pattern) changes in a face stimulus. Consistently, Sekuler et al. [44] found that upright and inverted face processings differ quantitatively, not qualitatively; in their study, observers utilized similar, local regions of faces for discrimination in both upright and inverted face conditions, and the relative contributions of perceptual mechanisms to performance were similar across orientations. As for the neural basis for such a mechanisms, Itier and Taylor [45] showed that the intracranial generators of N170 to inverted and upright faces came from the same lateral temporal region, near the superior temporal sulcus, and that the only difference between the two was the delayed response for inverted faces, thus supporting the hypothesis of a quantitative rather than qualitative difference between the two types of processing. In this context, subtraction between brain activations during perception of upright vs. inverted faces would not necessarily lead to the identification of the area devoted to face processing (or processing of configural analysis of faces).

The same line of reasoning might be applied to processing of words or other complex objects, recognizable by means of local and global features analysis.

In this regard, Bentin et al. [46] raise doubts about the assumption that inversion effects in general, and the face inversion effect in particular, exclusively reflect the disruption of configurational processes; rather, they may reflect a deficit in global processing not confined to faces. As for the possible right-sided lateralization of face recognition processes (within the right FFA), several ERP studies have indeed shown right lateralization of the N170 response to upright faces (e.g. [16]), which is thought to be the surface manifestation of Face Fusiform Area activity [46-48]. However, several papers have shown a lack of lateralization for the same stimuli [45], while others have shown a lack of inversion effect (upright/inverted discrimination [49]), or a right-sided lateralization that is not inversion specific [50-53]. Therefore, the question certainly deserves to be investigated further, not least in the light of possible gender differences in the degree of hemispheric lateralization, as advanced by Proverbio et al. [54].

The data of the present study evidenced a greater N1 to rotated than to standard words. The current literature shows the presence of a larger N1 to alphabetic than to non-alphabetic characters (e.g. [8,23,55]) during other implicit reading tasks. Other studies have shown an atypical/insufficient activation of VWFA as reflected by a lack of M170 increase in response to letters compared to icons in dyslexic individuals [56,57]. These data do not directly conflict with our results. In fact, the enhanced N1 response to mirror words may be interpreted as an index of unfamiliarity with rotated orthographic strings, rather than an enhanced response to non-alphabetic characters. The proof that rotated words are orthographic in nature (just not familiar) is given by the presence of implicit reading effects (i.e. words/pseudowords differences) for mirror words. In support of this interpretation, the fMRI study by Kronbichler et al. [31] describes an inverse relationship between frequency of words and degree of activation of the VWFA. Also, the review by Mechelli et al. [58] comparing brain activation in response to pseudowords and words found six out of nine studies in which pseudowords produced greater activation than words in regions corresponding to or near the VWFA. It is interesting to note that other studies have shown a similar inverse relationship (the greater the familiarity, the lower the activation) between object familiarity and degree of activation of left occipito-temporal regions (e.g. [29]). Again, there is evidence that N170 is larger in response to inverted than to upright faces [50,53], which supports our line of reasoning.

As for late semantic effects, the data from this experiment show how lexical access was delayed (by about 100 ms) but not disrupted for rotated words. This effect seemed to be restricted to the occipito/temporal regions since later anterior effects at the P300 or N400 levels were not observed. The data indicate that rotated words were discriminated from pseudowords within the ventral visual pathways (suggesting an invariant representation of words as perceptual objects), while there was a lack of explicit or attentive lexical access at later processing stages and more anterior brain areas. These data are consistent with the event-related fMRI findings of Bellgowan et al. [17] obtained with word and non-word stimuli rotated 0°, 60° or 120°. Their results showed that phonological processing areas (sensory-specific in nature equally to the left occipito and temporal regions in reading), such as the superior temporal and angular gyrus, were affected by lexical factors and showed no delay or width difference for rotated stimuli. On the other hand, the inferior frontal gyrus, which is involved in later lexical processing, showed a marked effect of stimulus inversion.

Overall, our behavioral and ERP data provided evidence of a strong 'words superiority effect' (e.g. [25,59]) in a paradigm not requiring semantic categorization, evident as faster or more efficient selection of an orthographic stimulus with an entry in the mental lexicon. In detail, our data showed faster RTs, fewer omission rates, greater posterior N2 and anterior P3 for words than for pseudowords. The lack of lexical effect earlier than 250, while reported by several ERP studies (e.g. [8,22,60,61]), has not been confirmed by other recent studies [25,26,59,62]. The relative timing inconsistency between studies can be attributed to various methodological parameters such as word length, word class, repetition rate, word frequency, display duration, task type, etc., as suggested by Martin et al. [59]. As for our specific task (involving target detection), it may be hypothesized that the presence of rotated words and pseudowords in an unblocked design might have induced the subjects to switch to a more local level of analysis than required by the same task for standard letters. The switch from a holistic to a more local type of analysis of string components might very likely have reduced the emergence of the earliest ERP effects of visual lexical recognition of familiar short words as global shapes.

## Competing interests

The author(s) declare that they have no competing interests.

## Authors' contributions

AMP conceived of the study, coordinated data acquisition and analysis, performed source localization, interpreted the data and drafted the manuscript. FW participated in the design of the study, collected data and performed statistical analyses. RA, VR and MDZ contributed to the ERP acquisition and analysis. AZ participated in the study design, performed source localization and worked on the manuscript.
